# *Games Wide Open* to athlete partnership in building artificial intelligence systems

**DOI:** 10.1038/s41746-024-01261-y

**Published:** 2024-10-01

**Authors:** Yosra Magdi Mekki, Osman Hassan Ahmed, Dylan Powell, Amy Price, H. Paul Dijkstra

**Affiliations:** 1 College of Medicine, Qatar University, Doha, Qatar; 2 The Football Association, St George’s Park, Burton-upon-Trent, UK; 3 University Hospitals Dorset NHS Foundation Trust, Poole Hospital, Poole, UK; 4 School of Sport, Health and Exercise Science, University of Portsmouth, Portsmouth, UK; 5 Faculty of Health Sciences & Sport, University of Stirling, Stirling, UK; 6 Dartmouth Institute for Health Policy & Clinical Practice (TDI), Geisel School of Medicine, Dartmouth College, Hanover, NH USA; 7 The British Medical Journal London, London, UK; 8 Department of Medical Education, Aspetar Orthopaedic and Sports Medicine Hospital, Doha, Qatar; 9 Nuffield Department of Orthopaedics, Rheumatology and Musculoskeletal Sciences, University of Oxford, Oxford, UK

**Keywords:** Institutions, Patient education

## Abstract

The integration of artificial intelligence (AI) in sports medicine is opening new frontiers for athlete health and performance, aligning with the spirit of the Paris 2024 Olympic Games slogan, “Games Wide Open.”

In the spirit of Paris 2024’s slogan, “Games Wide Open,” the integration of artificial intelligence (AI) in sports medicine aims to open new frontiers for athlete health and performance. Athletes and patients are increasingly recognized as essential partners in developing and implementing AI systems in sports medicine and science. AI systems in sports medicine have several possible applications, including diagnostic tools, behavior and performance monitoring systems, and external devices that monitor vital signs, sleep patterns, or other health metrics. These monitoring applications help in real-time assessment and management of an athlete’s health, enabling timely interventions and optimizing performance.

The Paris 2024 Olympics marks the first major influence of AI on the event, with several systems already in place^1^. For instance, the AthleteGPT chatbot provides athletes with round-the-clock assistance, answering questions and offering guidance through the Athlete365 app. Another significant AI application is Intel’s 3D Athlete Tracking (3DAT), which tracks athletes’ movements in real-time, offering biomechanical insights critical for optimizing performance and training. Additionally, a new AI-powered talent-spotting system is being tested to identify future Olympians by analyzing participants’ athletic potential through various physical assessments^2^. These AI systems are part of the broader AI agenda embraced by the International Olympic Committee (IOC), signaling a new era in athlete performance enhancement, injury prevention, and fan engagement.

In the Brisbane 2032 Olympics, AI could revolutionize the experience for athletes, coaches, team doctors, and fans by making return-to-play decisions more precise through predictive analytics, enhancing coach-athlete compatibility with AI-driven insights, and providing real-time health monitoring for team doctors to prioritize urgent issues. For fans, AI could personalize the viewing experience with tailored content, real-time stats, and augmented reality displays, creating a unique and engaging experience for every viewer.

These applications provide a lens for understanding the diverse applications of AI discussed throughout this paper. This paper was developed through a collaborative co-production process, involving clinicians, patients, and athletes, ensuring a comprehensive and multi-perspective approach to the integration of AI in sports medicine. Involving athletes as partners in the development, testing, and premarket evaluation of AI systems for sports medicine and performance is essential. Athletes provide practical insights into their specific needs and challenges, ensuring that the AI systems developed are both relevant and effective. Their direct feedback helps optimize the user interface, making these systems more intuitive and user-friendly. Additionally, athletes could contribute valuable performance and injury data, enhancing the accuracy and reliability of AI algorithms.

These AI systems can deliver significant value for athletes by monitoring and improving training, optimizing recovery strategies, and facilitating a more efficient return to play after injuries. By validating the efficacy and safety of AI systems in real-world conditions, we can ensure they meet the necessary standards before widespread implementation, ultimately enhancing athlete health and performance. This partnership also fosters trust and acceptance within the sports community, promoting wider adoption of AI systems. This strategy not only improves the athlete experience but also promotes inclusivity and broad partnership, aligning with Paris 2024’s vision of bringing the Olympic spirit closer to everyone. Consequently, the resulting systems are more responsive and better equipped to meet the unique needs of athletes and patients, leading to improved outcomes and greater acceptance. Moreover, partnership fosters a sense of ownership and empowerment, bolstering the successful adoption and integration of AI systems in sports and healthcare.

The 7th International Olympic Committee World Conference on Prevention of Injury and Illness in Sport^3^, a triennial event that took place from February 29 to March 2, 2024, emphasized the importance of involving athletes in co-producing AI systems tailored to their needs. This is in alignment with the Olympic AI agenda^4^, which highlights the significance of personalized athlete models and collaborative efforts across disciplines to enhance the practical application of AI in sports medicine. Under the direction of IOC President Thomas Bach, the Olympic AI Agenda is the third in a series of three strategy documents, and it details the anticipated effects that AI can bring to sports^4^.

Everyone involved in building AI systems should understand the contextual strengths, weaknesses, opportunities, and threats (SWOT) associated with AI in sports medicine and science research, coaching, and athletic performance optimization—this is essential for maximizing the benefits of AI whilst simultaneously mitigating potential risks^5^.

Accordingly, advancements in this area should prioritize partnering with athletes to ensure that AI systems are customized to meet individual requirements and foster a constructive healthcare provider-athlete relationship. The AI in Sports Medicine and Science Symposium held during the IOC Conference in Monaco highlighted practical steps in which AI could enhance shared decision-making between athletes/patients and experts from medical, science, and computational fields^6,7^. The session started with an introduction to the importance of AI in protecting athletes’ health. The essential role of AI and big data in preventing sports injuries and illnesses was outlined, followed by evidence supporting AI’s role in injury and illness prevention. The case for open-access regenerative AI to protect all athletes was advocated, and leading efforts in AI application in sports medicine were discussed.

Similar to other fields of medicine, shared decision-making is increasingly emphasized (and researched) in sports medicine, particularly in return-to-play decisions following injury^8^. This approach involves collaboration between the athlete, sports medicine clinicians, and other relevant parties (e.g. coaches or managers) to collectively deliberate on preferred decisions. The shift towards shared decision-making reflects a more athlete-centered approach in sports medicine, where the autonomy of the athlete is respected while considering the potential associated risks^9^. When athletes/patients are actively involved in developing, testing, and implementing AI (as in other systems), these interventions can be better tailored to specific needs, and enable systems to develop responsive tools that will be fit for purpose. Furthermore, qualitative research has concluded that athletes recognize the potential of AI-driven coaching technologies in improving training efficiency, personalization, and injury prevention^10^.

These systems (for example, AI chatbots for patient education) could make complicated clinical guidelines more approachable to end users, including athletes and clinicians. The IOC Conference showcased a range of posters and presentations on AI, highlighting the latest innovations and research in this field. They demonstrate how artificial intelligence and machine learning could augment sports medicine and accelerate the science to prevent injuries and illness, improve performance, and protect athletes’ health and well-being. One example of this was a poster estimating the injury risk of track and field athletes (practicing sports involving sprints) based on athletes’ physical and mental state perception monitoring^11^. Other outputs at the conference analyzed the frequency of view and the level of use of the injury risk estimation feedback with prognostic modeling approaches using machine learning techniques^12^.

Focusing on co-production, we propose five key aspects for the sporting community (Fig. 1) to focus on as we sail into unchartered AI waters (see below for more detail).

**Table:** 

Let Athletes Help!
1. Athlete, Clinician, and Patient Engagement: Involve us in every stage of AI development, from conception to evaluation. All data must be shared openly with end users.
2. Personalization: Design AI systems that create tailored models for individual athletes.
3. Adaptability: Develop “adaptive AI systems” that can evolve and adjust based on the changing needs and conditions of athletes over time.
4. Equity: Ensure AI systems are accessible and beneficial for all athletes, regardless of gender, culture, ability, or resources.
5. Innovation Ecosystems: Foster partnerships between researchers, startups, and established institutions to drive continuous advancement.

**Fig. 1:**
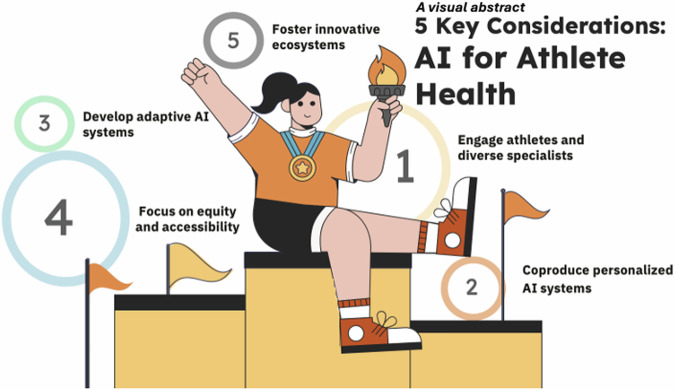
5 Key considerations when building AI for athlete health. A visual abstract displaying the five key considerations for the sporting community to consider when building AI solutions for the athlete population.

The further development of AI systems with an impact on sports medicine and science depends on active collaboration between athletes and interdisciplinary experts. Only when co-produced will AI systems truly tailor for the specific and highly contextual real-world needs of the sporting community. By focusing on personalized athlete models, promoting shared decision-making, and prioritizing accessibility and equity, the IOC and other national and international sporting bodies could create innovative, valuable, and practical AI systems. Not only would such efforts contribute to enhancing athlete health and performance, but also foster an inclusive and equitable environment, ultimately aligning with the Olympic AI agenda’s vision for the future of sports medicine. Embracing responsible AI practices is essential to bridging existing disparities and ensuring that the benefits of AI are distributed fairly, supporting all athletes, including those from minoritized groups.
