# Author Correction: *Games Wide* Open to athlete partnership in building artificial intelligence systems

**DOI:** 10.1038/s41746-024-01284-5

**Published:** 2024-10-19

**Authors:** Yosra Magdi Mekki, Osman Hassan Ahmed, Dylan Powell, Amy Price, H. Paul Dijkstra

**Affiliations:** 1 College of Medicine, Qatar University, Doha, Qatar; 2 The Football Association, St George’s Park, Burton-upon-Trent, UK; 3 University Hospitals Dorset NHS Foundation Trust, Poole Hospital, Poole, UK; 4 School of Sport, Health and Exercise Science, University of Portsmouth, Portsmouth, UK; 5 Faculty of Health Sciences & Sport, University of Stirling, Stirling, UK; 6 Dartmouth Institute for Health Policy & Clinical Practice (TDI), Geisel School of Medicine, Dartmouth College, Hanover, NH USA; 7 The British Medical Journal London, London, UK; 8 Department of Medical Education, Aspetar Orthopaedic and Sports Medicine Hospital, Doha, Qatar; 9 Nuffield Department of Orthopaedics, Rheumatology and Musculoskeletal Sciences, University of Oxford, Oxford, UK

**Keywords:** Institutions, Patient education

Correction to: *npj Digital Medicine* 10.1038/s41746-024-01261-y, published online 01 October 2024

In this article, the author name Dylan Powell was incorrectly written as Dyllan Powell. The original article has been corrected.

